# Supplementary material for: Engineering polar vortex from topologically trivial domain architecture
Source: Nat Commun. 2021 Jul 30;12:4620. doi: 10.1038/s41467-021-24922-y (PMC8324780; doi:10.1038/s41467-021-24922-y)
Supplement: Supplementary file 1 — Supplementary Information [file 41467_2021_24922_MOESM1_ESM.pdf]

Supplementary Information for

Engineering polar vortex from topologically trivial domain architecture

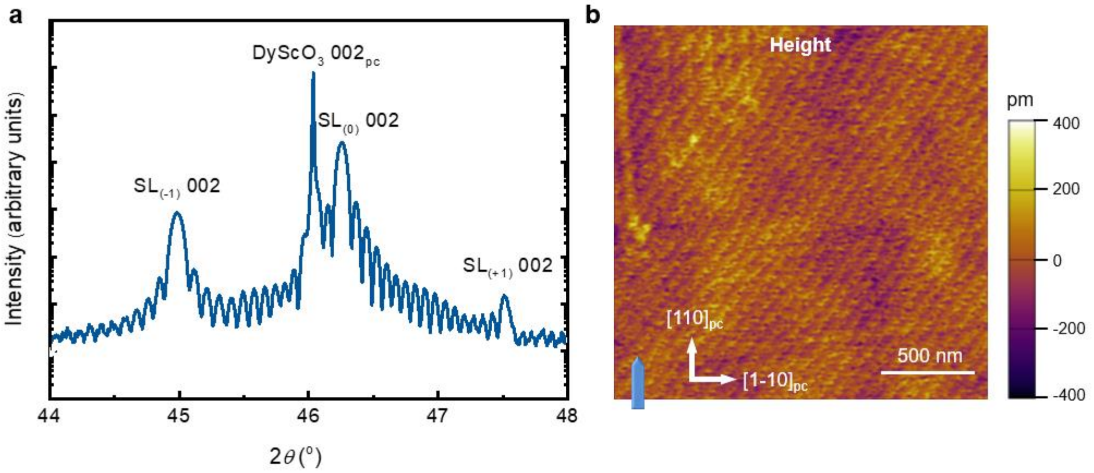

Supplementary Fig. 1 | a High-resolution θ - 2θ symmetric scan showing the presence of 15 *Pendellösung* fringes, which confirms the smoothness of the interface and the high quality of the $(PTO)_{10}/(STO)_{10}$ superlattice. **b** Surface morphology of 2×2 μm^2 area of the as-grown $(PTO)_{10}/(STO)_{10}$ surperlattice.

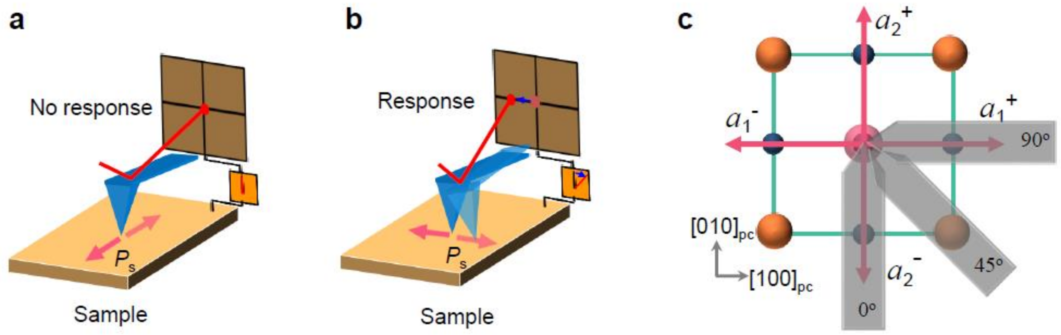

Supplementary Fig. 2 | Schematic of lateral piezoresponse force microscopy (LPFM). The scan angle was set to be 90°. **a** There is no response with polarization perpendicular to torsion direction. Red arrows represent in-plane ferroelectric polarization. **b** Lateral piezoresponse is induced with polarization component parallel to torsion direction. **c** Schematic view of the ferroelectric a_1/a_2 domain architecture with 90° domain walls. PFM tip cartoon display cantilever directions of the tip.

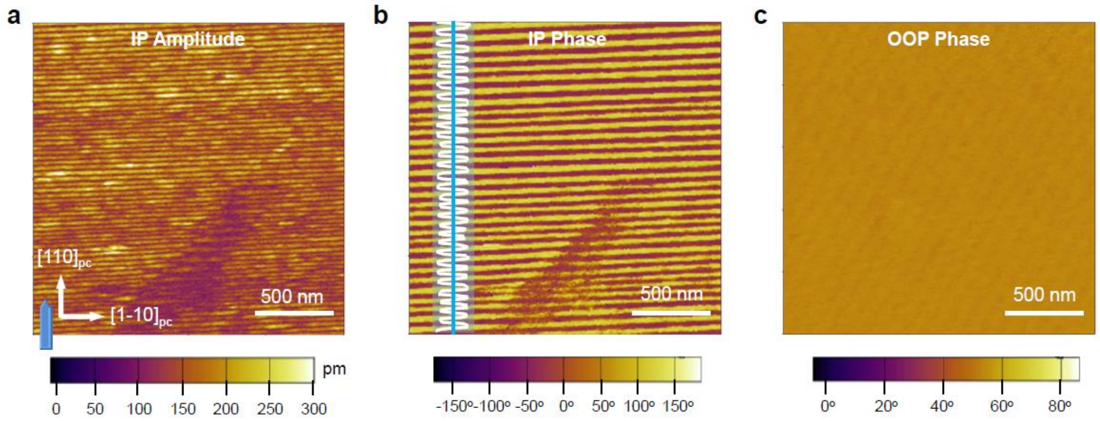

Supplementary Fig. 3 | a In-plane (IP) PFM amplitude image of 2 × 2 μm^2 area of the as-grown $(PTO)_{10}/(STO)_{10}$ superlattice shows parallel domain walls. **b** IP-PFM phase image shows stripe-like domain structure. Inset present the phase profile along blue line, showing in-plane phase difference in adjacent stripe is 180 degree. **c** Out-of-plane (OOP) PFM phase image with uniform contrast show no out-of-plane polarization.

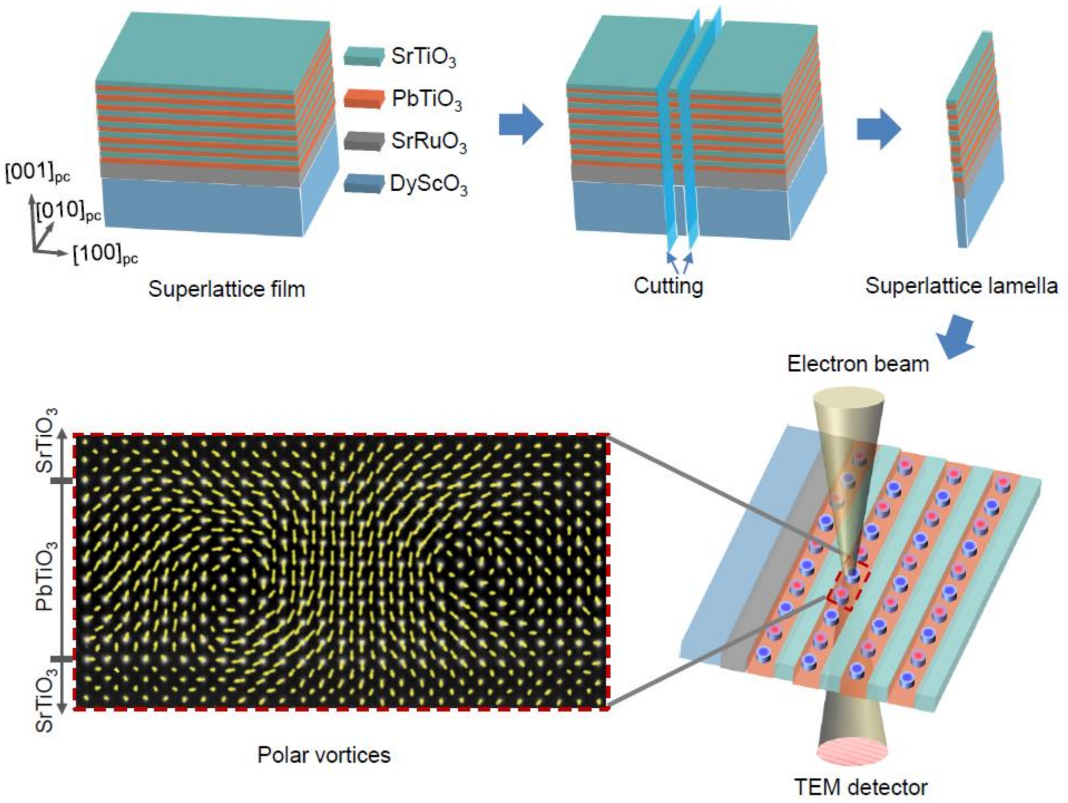

Supplementary Fig. 4 | Schematic of cutting TEM sample from $(PTO)_{10}/(STO)_{10}$ superlattice film.

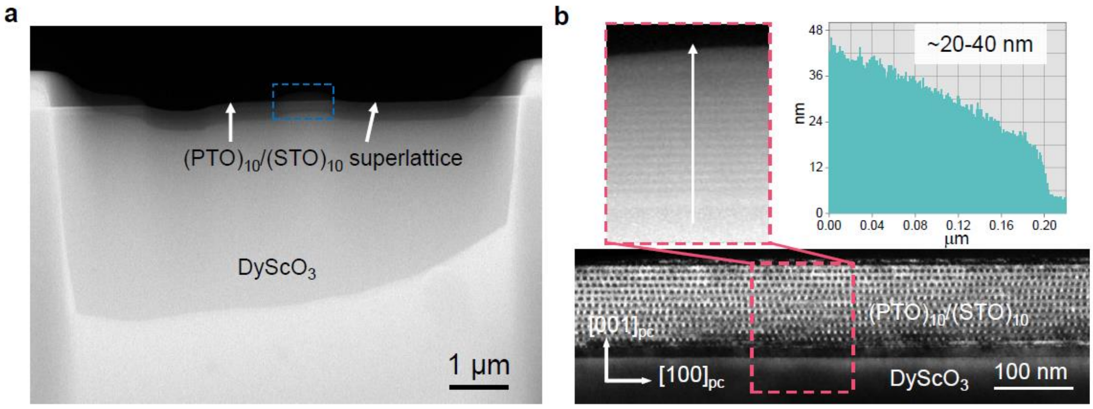

Supplementary Fig. 5 | a The lamella sample cut from superlattice film. **b** The DF-TEM image acquired from the blue box in (**a**). The upper left inset displays the STEM EELS thickness profile calculated using the log ratio method from a low-loss spectrum image. Line profile extracted at the white arrow showing that the thickness ranges from ~20 to ~40 nm.

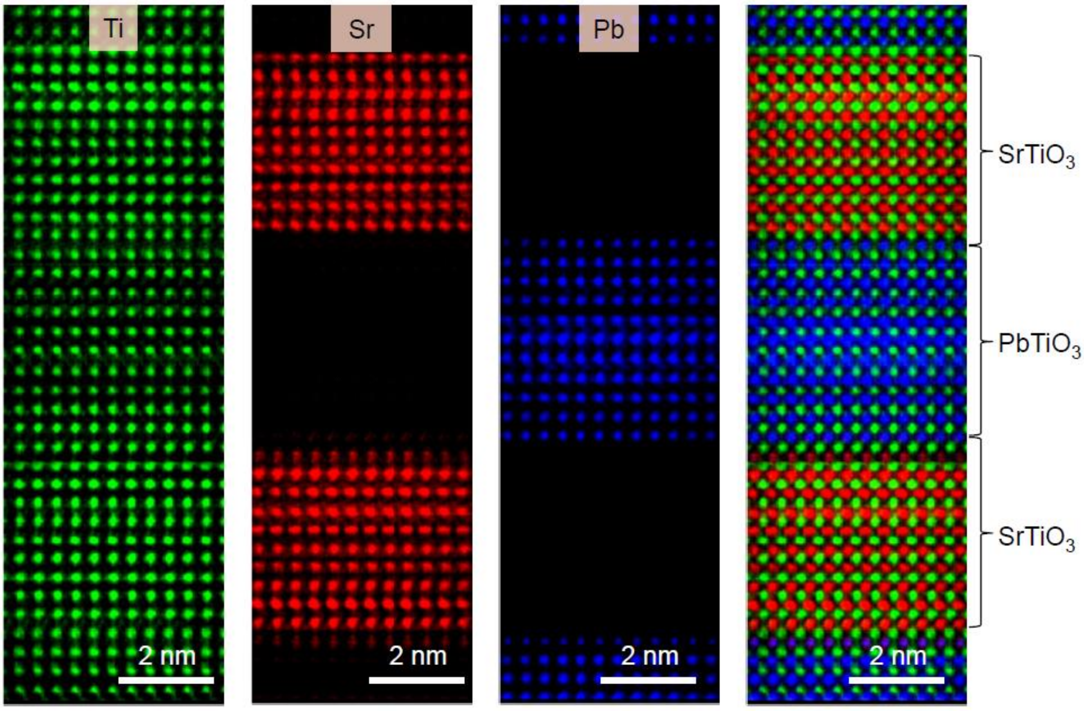

Supplementary Fig. 6 | Atomic EDS mapping of the $(PTO)_{10}/(STO)_{10}$ superlattice shows relatively sharp interface between $PbTiO_3$ and $SrTiO_3$.

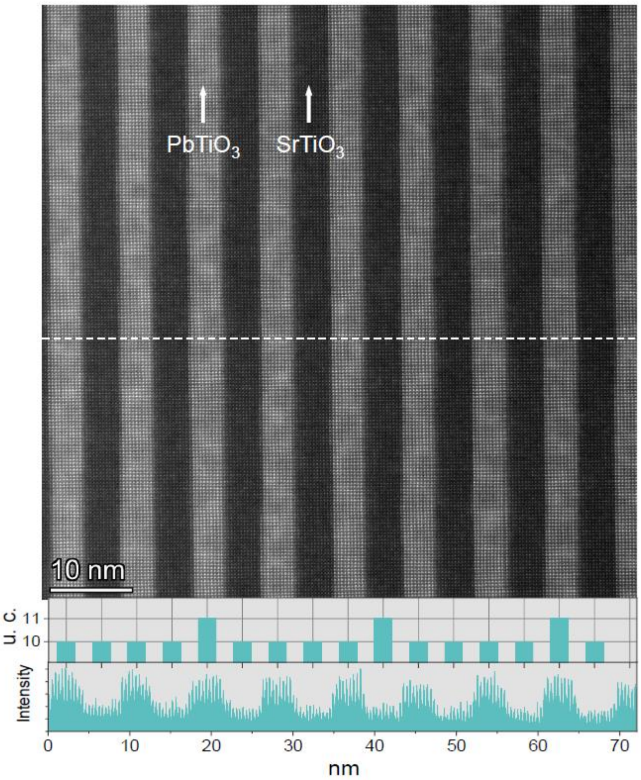

Supplementary Fig. 7 | ADF-STEM image of $(STO)_{10}/(PTO)_{10}$ superlattice and the corresponding line profile of the area labeled by the white short dash line, presenting the thickness of the PTO and STO layers in term of the number of unit cells (u. c.).

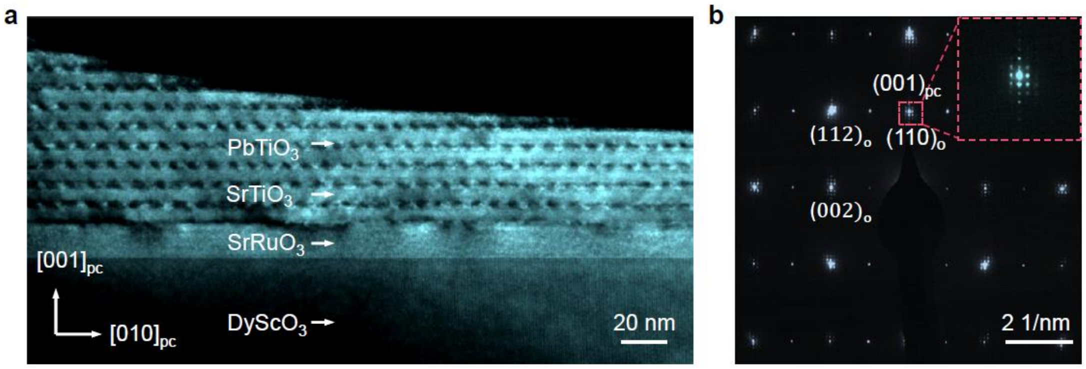

Supplementary Fig. 8 | a Cross-sectional DF-TEM image taken along the $[100]_{pc}$ (i.e. $[\bar{1}10]_{o}$) zone axis under two-beam condition by selecting 002_{pc} g-vector, with 20 nm scale bar. **b** The corresponding SAED pattern of (**a**) with enlarged $(001)_{pc}$ spots in the inset.

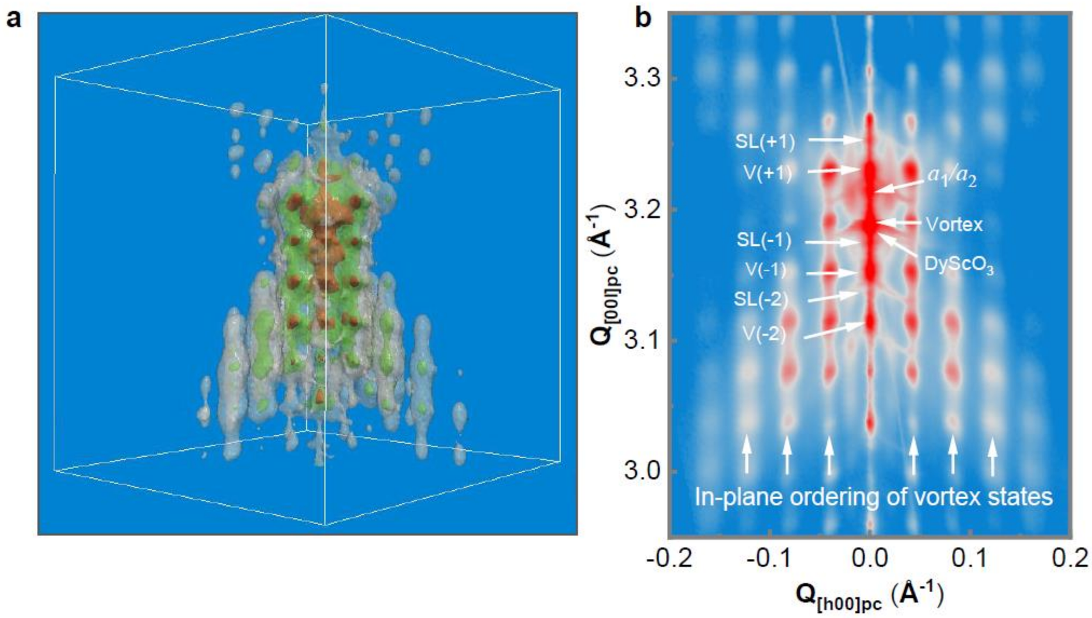

Supplementary Fig. 9 | a 3D-RSM around 002_{pc} Bragg spot of the $DyScO_3$ substrate with $[(PTO)_{20}/(STO)_{20}]_{17}$. **b** $Q_{[h00]}$-$Q_{[00l]}$ slice showing obvious in-plane satellite peaks with the same $Q_{[00l]}$ value beside reflection of PTO vortex phase and each of its superlattice reflections, such as marked vortex, v(+1), v(-1), v(-2), along the in-plane $[100]_{pc}$ direction with a periodicity of 15.6 nm. Meanwhile, the a_1/a_2 phase as well as its superlattice reflections could be recognized also in this figure without in-plane satellite peak, as marked as a_1/a_2, SL(+1), SL(-1), SL(-2).

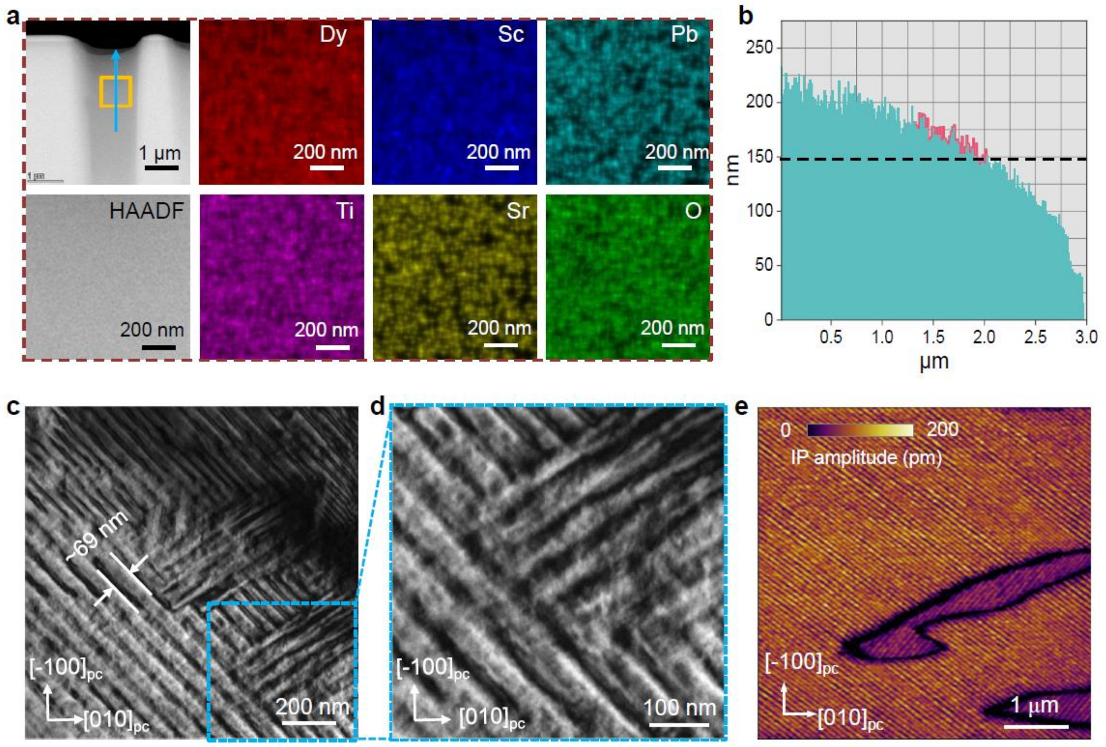

Supplementary Fig. 10 | a A planar-view STEM image of a $(PTO)_{10}/(STO)_{10}$ superlattice film and the EDS mapping corresponding to the marked yellow box showing uniform distribution of elements of PTO/STO superlattice film and the $DyScO_3$ substrate. **b** Thickness profile corresponding to the blue arrow in (**a**) extracted from the STEM EELS data, showing that the thickness for the orange box in (**a**) ranges from ~150 to ~180 nm (denoted by red line), exceeding the thickness of the $(PTO)_{10}/(STO)_{10}$ superlattice films. **c** The planar-view DF-TEM imaging for orange box area in (**a**) exhibits long-range in-plane ordering along $[110]_{pc}$ direction and confirms the periodic a_1/a_2 strip-like domain in the superlattice films. **d** The local-enlarged planar-view DF-TEM imaging in (**c**) shows a few stripe-like domain structure in perpendicular direction. **e** In-PFM amplitude image of 5 × 5 μm^2 area of the as-grown $(PTO)_{10}/(STO)_{10}$ superlattice shows that there are a small number of strip-like domains with perpendicular orientation as well, in good agreement with the DF-TEM results in (**c**) and (**d**).

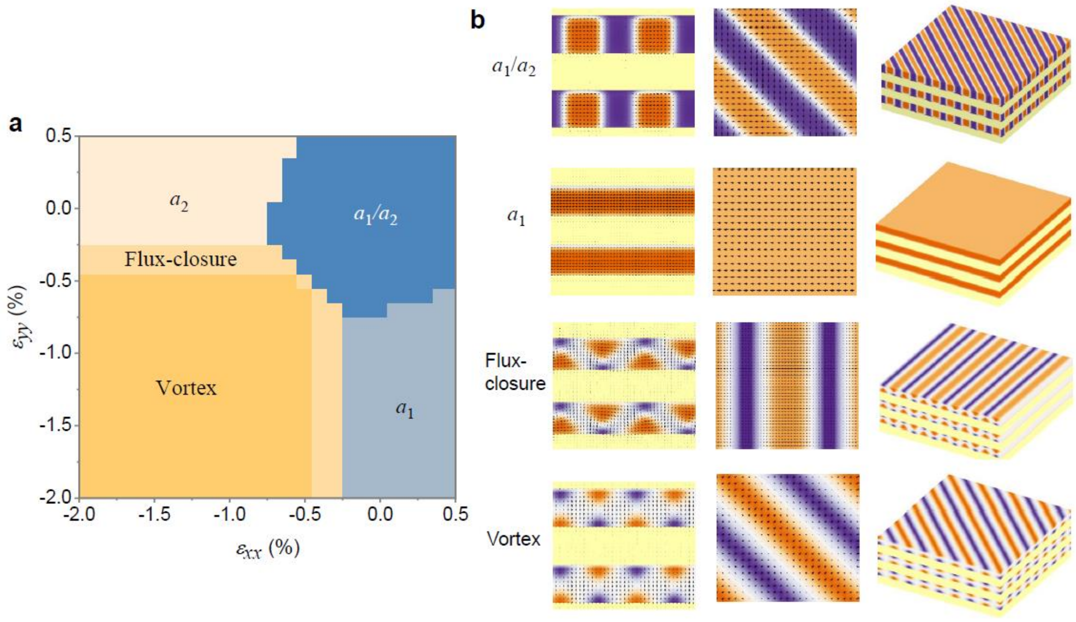

Supplementary Fig. 11 | a The $\varepsilon_{xx} - \varepsilon_{yy}$ phase diagram of the polar topological domain structure. **b** The local-enlarged domain architecture of a_1/a_2, a_1, flux-closure, and vortex phase at (110) and (001) plane, and the three-dimensional images, respectively.
